# Diabetic ketoacidosis treatment outcomes and its associated factors among adult patients with diabetes mellitus admitted to public hospitals in Nekemte Town, Ethiopia: a cross-sectional study

**DOI:** 10.3389/fcdhc.2024.1446543

**Published:** 2025-01-14

**Authors:** Daniel Mitiku Yigazu, Matiyos Lema, Firomsa Bekele, Dawit Tesfaye Daka, Dagim Samuel, Nigatu Addisu

**Affiliations:** ^1^ Department of Clinical Pharmacy, School of Pharmacy, Institute of Health Sciences, Wallaga University, Nekemte, Ethiopia; ^2^ Department of Public Health, Institute of Health Sciences, Wallaga University, Nekemte, Ethiopia; ^3^ Department of Pediatrics and Neonatal Nursing, School of Pharmacy Nursing and Midwifery, Institute of Health Sciences, Wallaga University, Nekemte, Ethiopia; ^4^ Department of Pharmacology, School of Pharmacy, Wallaga University, Nekemte, Ethiopia

**Keywords:** diabetic ketoacidosis, treatment outcome, associated factors, public hospitals, Nekemte Town

## Abstract

**Background:**

Diabetic ketoacidosis (DKA) is a serious and acute complication of diabetes mellitus. In Ethiopia, the mortality associated with acute diabetes complications ranges from 9.8% to 12%. Despite this, there is limited information on the clinical outcomes of DKA in our study location. Therefore, this study aimed to assess the magnitude and associated factors of DKA treatment outcomes among adult patients with diabetes admitted to public hospitals in Nekemte Town, Ethiopia.

**Objective:**

To assess the DKA treatment outcomes and their associated factors among adult patients with diabetes admitted to public hospitals in Nekemte Town.

**Methods:**

A 5-year cross-sectional study was conducted using a systematic random sampling technique among 201 patients from 1 July to 31 August 2023. DKA treatment outcomes were assessed at discharge. Pharmacists collected data by reviewing patient charts using Kobo Toolbox software. The data were then exported to SPSS Version 27 for analysis. Both bivariable and multivariable logistic regression analyses were performed. Variables with a P-value < 0.25 in the bivariable logistic regression were entered into the multivariable regression analysis to control for potential confounders. An adjusted odds ratio with a 95% confidence interval was used to identify predictors of treatment outcomes. A P-value < 0.05 was considered significant in the multivariable analysis.

**Result:**

Complete data was available for 201 patients admitted with DKA. The majority, 178 (88.6%), improved and were discharged. Independent predictors of DKA recovery were comorbidities [AOR: 3.45, 95% CI: 1.33, 9.72], admission Glasgow Coma Scale (GCS) score (<8) [AOR: 2.74, 95% CI: 1.02, 7.34], random blood glucose (RBS) (≥ 500) [AOR: 3.07 (95% CI: 1.12, 8.39)], and urine ketones (≥ +3) [AOR: 3.24, 95% CI: 1.18, 8.88].

**Conclusion and recommendation:**

Most of the treated patients with DKA were discharged with improvement. Comorbidity, admission GCS, RBS, and urine ketones were independently associated with DKA recovery. In general, significant consideration should be given to DKA prevention, early detection, and appropriate hospital management.

## Background

Diabetes mellitus (DM) is a diverse group of metabolic disorders that is characterized by persistently elevated blood glucose (1–6). According to statistics, the number of people with DM worldwide rose from 422 million in 2014 (7) to 463 million adults in 2019 (8) and 536.6 million people in 2021, and it is expected to reach 643 million in 2030 and 783.2 million in 2045 (9), indicating that the global prevalence of DM has reached pandemic proportions (10). In addition to this, the prevalence of DM in the African region was 4.7% in 2019; it is estimated to reach 5.1% and 5.2% in 2030 and 2045, respectively (9). Similarly, the International Diabetes Federation (IDF) (6) report in 2019 also shows that the national prevalence of DM in Ethiopia was 3.2% (2 million people) among adults aged 20-70 years. This places Ethiopia as the fourth-ranked country in terms of DM prevalence in Africa (8).

Diabetic ketoacidosis (DKA) is the most serious acute complication of DM (11, 12). It is a critical and potentially life-threatening complication that can arise in individuals with uncontrolled DM (13) and it can occur when there is an absolute or relative deficiency of insulin. It is more common in patients with type 1 DM, though it can also occur in patients with type 2 DM (14, 15). DKA is commonly precipitated by various factors, including infections, non-compliance with antidiabetic medications, and various stressors, such as cardiac ischemia, trauma, and pancreatitis (16).

Polyuria, polydipsia, weight loss, vomiting, dehydration, fatigue, altered mental status, tachycardia, and hypotension are among the clinical features of DKA (17). Fluid, electrolyte, and insulin therapy are used throughout treatment, coupled with careful evaluation and control of the precipitating factors (18, 19).

A report from Atlanta shows that among DM patients admitted with acute DM complications (ADMC), 38% had isolated DKA (20). A similar study conducted in China also showed that of the DM patients admitted, 41.1% had DKA (21). In addition to these, studies conducted in Ethiopia also reported that 26.4% of admitted DM patients developed ADMC. Of these, 73.9% had DKA (22). Similarly, a report from the Oromia region also shows that the prevalence of DKA accounted for 66.5% (23). The estimated mortality due to DM and its complications in 2021 was 6.7 million worldwide (9, 24), and there were 416,000 fatalities in 48 sub-Saharan African nations in 2021 (9). For instance, a study conducted in Cameroon showed that the overall case fatality rate due to DKA was 21.7% (25). Similarly, DKA mortality reports in Ethiopia also showed a 12% fatality rate in Shashemene (26) and 11.1% due to DKA in Harar (27). The potential factors for in-hospital mortality to DKA are lack of effective plasma osmolality (EPO) (21), hypoglycemia (26), advanced age, mechanical ventilation, bedridden state (28), and serum glucose fluctuation during hospitalization (29). The Centers for Disease Control also reported that between 2000 and 2014, hospitalizations for DKA rose by 6.3%/year in the USA (20).

The estimated global direct health expenditure on DM also increased from 232 billion in 2007 to USD 760 billion in 2019 and is expected to grow to a projected USD 825 billion by 2030 and USD 845 billion by 2045 (8, 30). According to estimates for 48 sub-Saharan African nations, African regions spent roughly 12.6 billion USD despite having 4.5% of all DM patients worldwide (9). Similarly, in Ethiopia over the past 20 years, DM placed a substantial economic strain on the country (31). For instance, a study showed that the mean monthly total cost of DM was US$ 37.7 (32).

To reduce this impact, different nations have implemented various methods and preventative measures, such as implementing DKA treatment recommendations and guidelines and educating people with DM about their condition (33). These strategies, however, have not been properly applied in Ethiopia. The prevention and management of DKA in Ethiopia is further complicated by the expense and scarcity of medicinal supplies, the presence of comorbid illnesses, medication non-adherence, electrolyte disturbance, and smoking behaviors (26, 34, 35).

Despite the significant challenges and costs associated with diabetes mellitus and its complications, studies on treatment outcomes and predictors among patients admitted with diabetes-related complications in Ethiopia are scarce. Moreover, the number of DKA patients and their impact on Ethiopian healthcare institutions is on the rise, necessitating more comprehensive studies in this area. In particular, there was no study available in the western part of Ethiopia that showed treatment outcomes and the factors associated with DKA. With the aforementioned facts in mind, this study aimed to assess treatment outcomes and their associated factors among DM patients admitted with DKA to public hospitals in Nekemte Town, Ethiopia, to provide input for patients, clinicians, researchers, and policymakers who want to advance better healthcare and reduce mortality from DKA.

## Methodology

### Study location, design, and period

A multiple-facility cross-sectional study was conducted at Nekemte Comprehensive Specialized Hospital (NCSH) and Wallaga University Referral Hospital (WURH) from 1 July to 31 August 2023.

### Study participants and eligibility criteria

All patients with DM who were admitted to NCSH and WURH with a diagnosis of DKA were included. Pregnant females admitted with DKA, patients with DKA with incomplete medical records, and patients with DKA and COVID-19 were excluded.

### Study variables and outcome endpoints

DKA treatment outcomes were the dependent variable. The socio-demographic characteristics of the patients, clinical characteristics, parameters and laboratory data measured at admission, comorbidities of the patients with diabetes, and precipitating factors for DKA-related admissions were the independent variables. DKA treatment outcomes were evaluated at discharge from the hospitals.

### Sample size and sampling technique

The single population proportion formula was used to calculate the required sample size by considering the following assumptions: n is the required sample size, and P is the proportion of patients discharged with an improvement of 73.8% (26). Z is the standardized normal distribution value at 95% CI= 1.96 and d is the margin of error of 5% (0.05).


$$\text{n}=\frac{{\left({\text{Z}}_{\text{α}/2}\right)}^{2}\text{p}\left(1-\text{p}\right)}{{\text{d}}^{2}}=\frac{1.96^{2}\left(0.738\right)*\left(1-0.738\right)}{0.05^{2}}=297$$

Since the sample was going to be taken from a relatively small population (<10000), with N= 500 being the total number of patients with DKA admitted within the last 5 years (June 01/2018 to May 30/2023), the sample size was adjusted as follows:


$$nf=\frac{n}{1+\frac{n}{N}}=186$$

where *n*=the desired sample, *n*f is the final sample size after adjustment. A 10% non-response rate was then accounted for and the final sample size was **205.**

**Figure 1:**
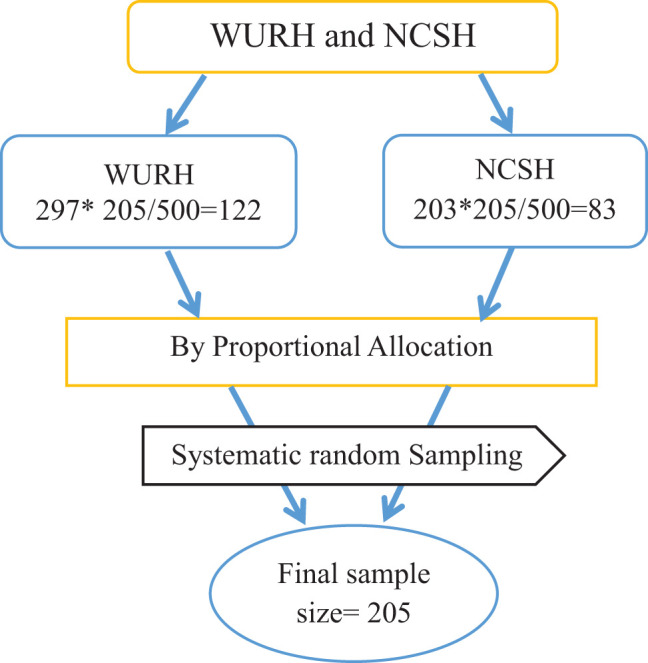
Schematic presentation of sampling technique to assess DKA treatment outcomes and their associated factors among adult patients with diabetes at WURH and NCSH.

The study used a systematic random sampling technique to select participants, with a K value of 2. The study participants were allocated proportionally for each hospital (**Figure 1**).

### Data collection process and management

The data extraction tool was pretested for the variables in the patient charts using 5% of the sample size (11 charts) at Ambo Referral Hospital. The tool was prepared by reviewing previously published literature (16, 29, 36, 37). Records of eligible patients were retrieved from the registration book. The medical registration numbers (MRNs) of all adult patients with DKA were then sorted. One in two charts (k = 2) was then randomly selected. After this, using the systematic random sampling method, subjects were selected, and data was collected using Kobo Toolbox software to ensure secure data collection, closely supervised by the lead investigators. Two licensed pharmacy professionals were selected for data collection, and one senior pharmacy professional was selected to supervise and organize the entire process during data collection for each hospital. Training was given to supervisors and data collectors.

### Data processing and analysis

Data inconsistencies, coding errors, completeness, clarity, missing values, and data cleaning were checked before exporting the data from Kobo Toolbox software to be analyzed by SPSS version 25. Descriptive statistics was conducted using percentages and frequency for categorical variables and mean and standard deviation for continuous variables. The multicollinearity of the independent variables was checked using the collinearity diagnostic test. The fitness of the regression model was checked using Hosmer–Lemeshow’s goodness of fit. The factors that predicted treatment outcomes were examined using bivariate and multivariate logistic regression, and variables with P-values less than 0.25 in the bivariable logistic regression analyses were candidates for the multivariable logistic regression analysis for controlling potential confounders. An adjusted odds ratio (AOR) with a 95% confidence interval (CI) was used to identify predictors of treatment outcomes in the final model. A P-value <0.05 was considered significant in the multivariable analysis. Finally, the processed data were presented by creating frequencies and percentages using tables, text, and graphs.

### Operational definitions and definitions of terms

- Comorbidity: the co-occurrence of one or more medical problems with diabetes.
- Diabetic ketoacidosis: defined based on a clinical diagnosis of DKA when there is random blood glucose (RBS) of > 250 mg/dl, urine ketones ≥ +2, arterial pH of < 7.3, and Bicarbonate ions of < 15 meq/l (38).
- Resolution of DKA: blood glucose <200 mg/dl and a negative dipstick for urine ketones for at least two successive measurements two hours apart after treatment interventions (39).
- Treatment outcomes: refers to outcomes following treatment intervention(s); showing improvement and discharged or died while receiving treatment.

## Results

### Socio-demographic characteristics of the study subjects

The study included 201 DKA patients with complete cards (98.05% response rate). Among them, 114 (56.7%) were men, 86 (42.8%) had a secondary education, and 93 (46.3%) were in the 31–40 age range. The mean age of the participants was 35.49 ± 14.81 (range 18–75 years). More than half of them (109, 54.2%) were urban residents and 140 (69.7%) were from the Oromo ethnic group. Protestants, (124, 61.7%), farmers, (76, 37.8%), and being married (130, 64.7%) constituted the majority of the patients. Of the reviewed patients, less than half (94, 46.8%) had health insurance coverage (**Table 1**).

**Table 1:** Socio-demographic characteristics of patients with DKA admitted to WURH and NCSH, from 1 June 2018 to 30 May 2023.

Variable	Category	Frequency (n) and (%)
Age	18-30	36 (17.9)
	31-40	93 (46.3)
	41-50	37 (18.4)
	51-60	24 (11.9)
	>60	11 (5.5)
Gender	Men	114 (56.7)
	Women	87 (43.7)
Area of residence	Urban	109 (54.2)
	Rural	92 (45.8)
Educational status	No formal education	25 (12.4)
	Primary school	70 (34.8)
	Secondary school	86 (42.8)
	Higher education	20 (10.0)
Marital status	Married	130 (64.7)
	Single	71 (35.3)
Ethnicity	Oromo	140 (69.7)
	Amhara	57 (28.4)
	Gurage	4 (2.0)
Religion	Protestant	124 (61.7)
	Orthodox	47 (23.4)
	Muslim	30 (14.9)
Occupation	Farmers	76 (37.8)
	Student	40 (19.9)
	Merchant	35 (17.4)
	Government employee	15 (7.5)
	NGO employee	5 (2.5)
Health insurance coverage	Yes	94 (46.8)
	No	107 (53.2)

(n=201).

### Clinical characteristics of patients with DKA

Of the reviewed patients’ cards, 41 (20.4%) had a family history of DM. In addition to this, 86 (42.8%) had newly diagnosed DM, and 44 (21.9%) had a comorbidity. More than half of the patients had DM for less than 8 years (83, 72.2%). Hypertension, congestive heart failure (CHF), and HIV were documented in 24 (54.5%), 17 (38.6%), and 3 (6.8%) patients, respectively.

More than half of the patients (105, 52.2%) had a history of being readmitted to the hospital since diagnosis. Out of these, 93 (46.3%) had previous readmissions in the last year. The frequency of DKA recurrence since diagnosis was also assessed and most of the patients had multiple episodes of DKA, with 24 (11.9%), 60 (29.9%), and 117 (58.2%) patients having one, two, and ≥ 3 episodes of DKA, respectively (**Table 2**).

**Table 2:** Clinical characteristics of the patients with DKA admitted at WURH and NCSH from 1 June 2018 to 30 May 2023.

Variable	Variable category	Frequency (n) and (%)
Readmission	Yes	105 (52.2)
	No	96 (47.8)
Previous admission due to DKA in the last 1 year	Yes	93 (46.3)
	No	108 (53.7)
Number of DKA episodes	Once	24 (11.9)
	Twice	60 (29.9)
	≥ Three times	117 (58.2)
Family history of DM	Yes	41 (20.4)
	No	160 (79.6)
Newly diagnosed DM	Yes	86 (42.8)
	No	115 (57.2)
Comorbidity	Yes	44 (21.9)
	No	157 (78.1)
Type of comorbidity	Hypertension	24 (54.5)
	CHF	17 (38.6)
	HIV	3 (6.8)
Type of DM	Type I	111 (55.2)
	Type II	90 (44.8)
Number of years since diagnosis	<8	83 (72.2)
	>=8	32 (27.8)

(N=201).

### Clinical characteristics of DKA patients at admission

Most of the patients (133, 66.2%) had a GCS score between 9 and 15 at admission, and the remaining 68 (33.8%) had a lower GCS score (3–8). Regarding urine ketones at admission, 118 (58.7%) were <+3 while the remaining 83 (41.3%) had ≥ +3. In addition to this, 86 (42.8%) had plasma RBS at admission of ≥500 mg/dl. Nearly two-thirds (130, 64.7%) of the admitted patients had normal blood pressure (BP) at admission to the hospitals. More than half (58, 50.4%) of the patients were on insulin treatment, 47 (40.9%) were receiving oral treatment, and 10 (8.7%) patients were on both oral and insulin treatments.

At the time of admission, 27 (27.6%) and 36 (34.3%), had hyperkalemia (serum potassium (K^+^) ≥ 5.2 mEq/L) and hypernatremia (serum sodium (Na^+^) ≥ 145 mEq/L) respectively. Additionally, 10 (9.8%) and 34 (43.6%) patients had elevated admission serum creatinine (SCr) (≥ 1.2 mg/dL) and blood urea nitrogen (BUN) (≥ 20 mg/dl), respectively **Table 3**.

**Table 3:** Admission clinical characteristics of the patients with DKA admitted at WURH and NCSH from 1 June 2018 to 30 May 2023.

Variable	Variable category	Frequency (n) and (%)
Urine ketones	<3	118 (58.7)
	>=3	83 (41.3)
GCS score	3-8	68 (33.8)
	9-15	133 (66.2)
Random blood glucose at admission	<500	115 (57.2)
	>=500	86 (42.8)
Blood pressure (BP)	Hypotension	13 (6.5)
	Normal BP	130 (64.7)
	Elevated BP	29 (14.4)
	Stage I hypertension (HTN)	11 (5.5)
	Stage II HTN	16 (8.0)
	Hypertensive emergency/urgency	2 (1.0)
Medication	Insulin only	58 (50.4)
	Oral medication only	47 (40.9)
	Both insulin and oral medications	10 (8.7)
Serum potassium (mEq/L)	<3.5	16 (16.3)
	3.5-5.2	55 (56.1)
	>5.2	27 (27.6)
Serum sodium (mEq/L)	<135	21 (20.0)
	135-145	48 (45.7)
	>145	36 (34.3)
Serum chloride (mEq/L)	<95	2 (2.2)
	95-107	46 (50.0)
	>107	44 (47.8)
Serum creatinine (mg/dL)	<0.5	12 (11.8)
	0.5-1.2	80 (78.4)
	>1.2	10 (9.8)
Blood urea nitrogen (mg/dL)	<7	2 (2.6)
	7-20	42 (53.8)
	>20	34 (43.6)

(N=201).

### Treatment outcomes of patients with DKA admitted to WURH and NCSH

**Figure 2:**
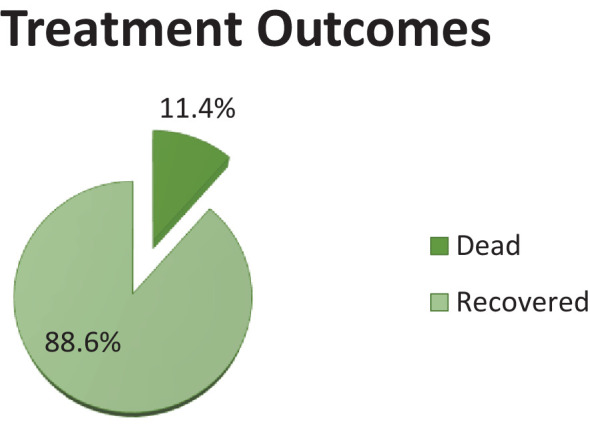
Treatment outcomes of patients with DKA admitted to WURH and NCSH.

Of the total number of participants, 178 (88.6) recovered from DKA and were discharged and 23 (11.4%) died while being treated during their hospital stay (**Figure 2**).

### Factors associated with DKA treatment outcomes

Binary logistic regression was used to examine the factors that were significantly associated with the treatment outcomes of DKA. It indicated that area of residence, newly diagnosed DM, precipitating factors, comorbidity, GCS score, urine ketones at admission, and history of readmission were variables that fulfilled the assumption (p < 0.25) and were considered candidates for the multi-variable analysis. In the multivariable logistic regression analysis, the presence of comorbidity, RBS level, GCS score, and ketones at admission had a statistically significant association with a good treatment outcome (recovery) after controlling for potential confounders.

Patients who had no comorbidities were approximately 3.49 times more likely to recover compared to those who had comorbidities [AOR = 3.49, 95% CI: 1.34, 9.72]. However, patients who had a higher GCS score at the time of admission (GCS ≥ 9) were 2.74 times more likely to recover compared to those who had a lower GCS score at the time of admission (GCS ≤ 8) [AOR = 2.74, 95% CI: 1.02, 7.34]. Patients who had lower RBS levels (< 500) at the time of admission were 3.07 times more likely to recover compared to those who had higher RBS levels (≥ 500) [AOR = 3.07 (95% CI: 1.12, 8.39]. Those who had urine ketones < +3 at the time of admission were approximately 3.24 times more likely to recover compared to those who had urine ketones ≥ +3 [AOR = 3.24, 95% CI: 1.18, 8.88]; (**Table 4**).

**Table 4:** Bivariable and multivariable Regression analyses of factors associated with DKA treatment outcomes in patients admitted to WURH and NCSH, from 1 June 2018 to 30 May 2023.

Variable	Category	Treatment outcome		COR(95%CI)	AOR(95%CI)	P-value
		Died	Recovered			
		N (%)	N (%)			
Place of residence	Urban	8 (34.8)	101 (56.7)	2.46 (0.99, 6.10)	2,24 (0.787, 6.40)	0.130
	Rural	15 (65.2)	77 (43.3)	1	1	
Readmission	Yes	17 (73.9)	88 (49.4)	1	1	
	No	6 (26.1)	90 (50.6)	2.89 (1.09, 7.69)	2.62 (0.88.7.78)	0.084
Newly diagnosed DM	Yes	11 (47.8)	75 (42.1)	1	1	
	No	12 (52.2)	103 (57.9)	1.26 (.53-3,01)	1.13 (0.41, 3.06)	0.817
Presence of comorbidity	Yes	12 (52.2)	32 (18.0)	1	1	
	No	11 (47.8)	146 (82.0)	4.98 (2.02, 12.30)	3.49 (1.33, 9.72)	0.012*
GCS score	3-8	13 (56.5)	55 (30.9)	1	1	
	9-15	10 (43.5)	123 (69.1)	2.91 (1.20, 7. 03)	2.74 (1.02, 7.34)	0.045*
RBS level at admission	<500	8 (34.8)	107 (60.1)	2.83 (1.14, 7.01)	3.07 (1.12, 8.39)	0.029 *
	>=500	15 (65.2)	71 (39.9)	1	1	
Urine ketones at admission	<3	8 (34.8)	110 (61.8)	3.03 (1.22, 7.53)	3.24 (1.18, 8.88)	0.022*
	>=3	15 (65.2)	68 (38.2)	1	1	

* P-Value is significant at ^*^ p <0.05.

## Discussion

Among the 201 DKA patients included in the study, a significant proportion of individuals, (178, 88.6%) [95% CI: 84.1, 92.9], experienced a recovery, while 23 died (11.4%) [95% CI: 7.1, 15.9]. This recovery rate is notably higher compared to previous studies conducted in other countries. For instance, the recovery rates reported in Malaysia (40), Nigeria (41), and Kenya (42) were 82.4%, 82%, and 70.2% respectively. A possible reason for the difference may lie in the difference in patient demographic and clinical characteristics such as the patient’s admission features and precipitating condition for DKA. For instance, in a study conducted in Kenya, over 90% of participants had altered consciousness, over 25% were in a coma, 36% had systolic hypotension, and nearly 75% had moderate to severe dehydration; and in the Nigerian subjects, older age (mean age 53.9 years) was more common than in this study.

However, the number of patients who recovered was relatively comparable with the findings in Shashemane (88%) (36), Harar (89.9%) (43), Adama (84.9%) (37), and Jimma (90.1%) (36). However, the percentage of patients recovered in the present study was lower than in studies conducted in Saudi Arabia (98.7%), Gondar (95.6%), Spain (98.8%), Thailand (98.17%), and Canada (95.7%) (16, 29, 36, 44–47). The higher percentage of recovery in Saudi Arabia, Spain, Thailand, and Canada might be due to differences in the quality of care provided in each hospital, health-seeking behavior, time from symptom onset to hospital visit, the inpatient management protocol for DKA, the availability of adequate laboratory investigations and monitoring during treatment to monitor patient response, and the accessibility of different medications. However, the disparity between reports from Ethiopia, i.e. Gondar, could be attributed to a lack of appropriate DM care, and financial limitations for laboratory support and medication, as it was found that in the majority of patients, DKA was brought on by non-compliance with their medication due to an issue with affordability and unavailability.

In this study, non-compliance to antidiabetic medications (51.5%) due to unaffordability was the most common, followed by infections (39.4%). This was also reported in other studies that were conducted in Shashemane (26), United Arab Emirates (48), Saudi Arabia (49), and Brazil (50), which showed a similar pattern as non-compliance to antidiabetic medication was the most common among patients admitted with DKA. However, this is in contrast with worldwide reports that the most common precipitating factor for DKA is infection followed by non-compliance to antidiabetic medication (51, 52). In the same way, research from Jimma (36), Cameroon (25), Tanzania (53), and Pakistan (54, 55) also indicates that infection is the most typical cause of DKA. This variation in precipitating factors was attributed to the differences in populations across the world (52).

The high incidence of patients who had previously received treatment (57.2%) may also be responsible for this study’s higher rate of non-compliance. Non-compliance with antidiabetic drugs in tropical and sub-Saharan Africa was also more common due to the cost and use of alternative remedies including herbs and prayers (2, 56). In addition to this, according to another study, widespread poverty among both people and countries in Sub-Saharan Africa makes healthcare services scarce (2). Infection prevention practices in Ethiopia are also poor (57). Hence, this study’s greater rate of infection (43.4%) as a precipitant after non-compliance may have been due to underlying poverty and poor infection prevention techniques. It is also conceivable that because 42.8% of the patients had diabetes for the first time, they may not have had much exposure to infection control and self-care (as known DM patients will be informed on such issues).

In this study, patients with DKA who had no comorbidities recovered more frequently than those who had comorbidities. Research from Harar (43), Jimma (36, 58), Shashemane (26), and Italy (59) also revealed this. This might be a result of comorbidities impacting several body systems, which can worsen a disease’s severity, make its clinical course more complex, and impair the body’s natural defenses against illness. Comorbidities can also further impair organ function and make it more difficult to follow the recommended course of treatment for DKA, which can ultimately result in a reduced rate of recovery.

In this study, DKA patients who had an RBS level < 500 mg/dl recovered more frequently than those who had an RBS level ≥ 500 mg/dl. The research conducted in Debre Markos supports this finding (60). An additional study that showed early blood glucose levels were a good predictor of rapid remission in patients with DKA also lends weight to this finding (61). This could be a result of excessively high blood glucose levels, which can lead to loss of blood volume, low blood pressure, and impaired central nervous system function (hyperglycemic coma), all of which can reduce the likelihood of recovery.

In addition, patients with DKA who had urine ketones < +3 recovered more frequently than those who had urine ketones ≥ 3. This finding was supported by a study conducted in the USA (62). This could be explained by the fact that elevated blood ketone levels may be linked to high blood glucose in this study, which may also contribute to increased blood acidity, the risk of complications including organ damage, and a decreased chance of recovery for the patients.

This study also showed that patients with DKA who had a GCS score < 8 recovered less frequently than those who had a GCS score ≥ 8. This finding is in line with studies conducted at Hiwot Fana Specialized University Hospital (43) and in Zambia (63), India (64), and Indonesia (65) where lower admission GCS scores (≤ 8) were the independent predictors of mortality among hospitalized patients with DKA. This is explained by the fact that comatose people are more likely to experience aspiration with DKA. Additionally, since most of these patients’ mental states were altered at the time of presentation, the likelihood of organ failure is also significant, which raises the possibility of death.

### Strengths and limitations of the study

This study was limited to secondary data. Thus, the analysis of the factors associated with treatment outcomes of DKA was based on information that was obtained from charts. For instance, it did not include information related to economic status, level of physical exercise (PE), BMI, and social drug use.

Despite its limitations, the study provides valuable insights that can directly impact patient care and clinical protocols in these settings. Identifying non-compliance, infections, and new-onset diabetes as the most common precipitants of DKA is crucial for understanding the local context and developing targeted preventive strategies. The identification of factors including comorbidity, GCS score, RBS level, and urine ketones that were independently associated with DKA recovery also provides valuable clinical predictors.

## Conclusion and recommendations

### Conclusion

The majority of patients with DKA treated at WURH and NCSH were discharged after attaining recovery. The main precipitating factor of DKA was non-compliance, which was followed by infection and newly diagnosed DM. The independent predictors of recovery were comorbidity, GCS score > 8, RBS level ≤ 500, and ketones ≤ +3. An intervention to reduce morbidity and mortality among patients with DM admitted for DKA should develop strategies that specifically target these modifiable predictive factors of mortality. In general, significant consideration should be given to DKA prevention, early detection, and appropriate hospital management.

### Recommendations

Based on these findings, we would like to recommend the following:

- Physicians should detect and manage precipitants of DKA as early as possible at the initial patient presentation at the hospital.
- Physicians should be aware of and treat patients who have independent predictors such as lower GCS scores, elevated ketones, and higher RBS levels.
- Healthcare providers and diabetes associations should promote education and improve self-care.
- Infection prevention strategies should be designed and implemented by diabetes patients, diabetes associations, the general public, healthcare providers, and the government to reduce infection-related admissions of DKA.
- Finally, we recommend large-scale multicenter prospective studies on diabetes in general, and DKA in particular, be conducted to assess the limitations in diabetes care in Ethiopia and devise strategies for cost-effective and evidence-based care of patients with this disease.

## Data Availability

The materials used while conducting this study are obtained from the corresponding author on reasonable request. Requests to access these datasets should be directed to danielmitiku38@gmail.com.
